# Flowing Liquid-Based Triboelectric Nanogenerator Performance Enhancement with Functionalized Polyvinylidene Fluoride Membrane for Self-Powered Pulsating Flow Sensing Application

**DOI:** 10.3390/polym16040536

**Published:** 2024-02-16

**Authors:** Duy Linh Vu, Quang Tan Nguyen, Pil Seung Chung, Kyoung Kwan Ahn

**Affiliations:** 1 Department of Nanoscience and Engineering, Inje University, 197 Inje-ro, Gimhae-si, Gyeongsangnamdo 50834, Republic of Korea; vuduylinhbk@gmail.com; 2 School of Mechanical Engineering, University of Ulsan, 93 Daehak-ro, Nam-gu, Ulsan 44610, Republic of Korea; pax.quangtan@gmail.com; 3 Department of Energy Engineering, Inje University, 197 Inje-ro, Gimhae-si, Gyeongsangnamdo 50834, Republic of Korea

**Keywords:** fluid-based triboelectric nanogenerator, pulsating flow rate, fluorinated functionalized, polyvinylidene fluoride, self-powered sensor

## Abstract

Pulsating flow, a common term in industrial and medical contexts, necessitates precise water flow measurement for evaluating hydrodynamic system performance. Addressing challenges in measurement technologies, particularly for pulsating flow, we propose a flowing liquid-based triboelectric nanogenerator (FL-TENG). To generate sufficient energy for a self-powered device, we employed a fluorinated functionalized technique on a polyvinylidene fluoride (PVDF) membrane to enhance the performance of FL-TENG. The results attained a maximum instantaneous power density of 50.6 µW/cm^2^, and the energy output proved adequate to illuminate 10 white LEDs. Regression analysis depicting the dependence of the output electrical signals on water flow revealed a strong linear relationship between the voltage and flow rate with high sensitivity. A high correlation coefficient R^2^ within the range from 0.951 to 0.998 indicates precise measurement accuracy for the proposed FL-TENG. Furthermore, the measured time interval between two voltage peaks precisely corresponds to the period of pulsating flow, demonstrating that the output voltage can effectively sense pulsating flow based on voltage and the time interval between two voltage peaks. This work highlights the utility of FL-TENG as a self-powered pulsating flow rate sensor.

## 1. Introduction

In recent times, the rapid expansion of the Internet of Things (IoT) has led to a prevailing trend in the development of electronic technology, specifically focusing on miniaturized and portable devices. This trend underscores a growing interest in self-powered functionality, aiming to extend operational durations while reducing reliance on conventional battery usage [1,2,3,4]. Consequently, the exploration of micro/nano-technology has captivated the attention of numerous researchers, culminating in the creation of nanogenerators grounded in piezoelectric, pyroelectric, thermoelectric, and triboelectric principles [5,6,7,8,9]. Since it was invented in 2012, the triboelectric nanogenerator (TENG) has emerged as a prominent technology for harvesting ambient mechanical energy from various environmental sources, including vibrations, human motion, wind, and ocean waves [10,11,12,13]. The working mechanism of TENG relates to the coupling of contact electrification and electrostatic induction between diverse materials—solid, liquid, and gas [14,15,16]. TENG devices have undergone rapid development, showcasing the significant potential for harvesting energy from low-frequency mechanical sources [9,17,18,19]. 

Notably, TENGs have evolved into smart sensing devices, uniquely influencing input parameters and generating corresponding electrical responses. Their attributes include self-powered operation, cost-effectiveness, simple structure, easy fabrication, portability, and high reliability [20,21,22,23]. Furthermore, water-based TENGs have gained attention due to the adaptable nature of water, enabling effective contact with solid layers for enhanced triboelectric charge generation. Several studies have investigated the use of water-based TENGs in sensing devices, including those for detecting tubular flowing water, liquid level, humidity, and chemical detection [23,24,25,26]. However, limited attention has been given to the study of unsteady flow, primarily due to the intricate nature of flow dynamics. Therefore, it is necessary to conduct research on water-based TENGs for converting energy from water movement into electricity and employ them as sensing devices to measure the flow rate.

Pulsating flow is commonly used to characterize a specific type of flow, characterized by the combination of a periodically oscillating flow and a steady flow [27,28]. This concept holds significant relevance in various industries and medical fields, with applications spanning heat transfer augmentation, improved cleaning processes, fluid mixing, mass transport in porous media, and biofluid engineering [29,30,31]. Pulsating flow in pipes has been observed in diverse technical areas such as physiology, roller and finger pumps, transportation of blood flow, oxygen, and sanitary fluids, among others [32,33,34,35,36,37]. Scientists have shown considerable interest in studying pulsating flow, especially focusing on accurate flow rate measurement, which is crucial for evaluating system performance [38,39,40]. Typically, flow rate determination involves multiplying physical quantities by correction factors corresponding to measurement technologies like turbine rotational frequency [40], pressure drops through an orifice [41], electromotive force [42], ultrasonic wave transit time, or Doppler effect [43]. However, these technologies have their disadvantages; for instance, flow disturbance, high cost, complex structure, and limited application in millimeter-scale pipelines. Consequently, the flowing liquid-based TENG (FL-TENG) emerges as a promising solution to address these limitations and serve as a self-powered pulsating flow sensor. The success of FL-TENGs in practical applications relies on their ability to generate sufficient output power, usually stored in a capacitor or battery. Nevertheless, the suboptimal quality of materials used to make FL-TENGs degrades their output performance, making them unsuitable for powering electronic devices [44,45]. Therefore, there is a need to enhance the performance of FL-TENGs by increasing the transferred charge density through liquid–solid electrification. In our prior studies, we thoroughly investigated the effect of fluorinated functionalization on the output voltage and current of TENG devices utilizing a PVDF membrane. The application of functionalization was found to significantly influence the output performance of the TENG device [44,46].

This paper introduces an inventive and advanced approach to measuring pulsating flow in pipelines, utilizing a FL-TENG that exhibits heightened output performance due to the integration of a functionally enhanced triboelectric layer. To obtain a high-charge transferred density triboelectric layer, we employed a fluorinated functionalized technique on a polyvinylidene fluoride (PVDF) membrane. This involved grafting the membrane with negatively charged 1H,1H,2H,2H-Perfluorooctyltrie-thoxysilane (FOTS). The substantial negative polarizations of fluorine played a pivotal role in significantly improving the dielectric constant and the hydrophobic property of the functionalized PVDF (F-PVDF) membrane, which led to a notable increase in the performance of the TENG. The F-PVDF-based TENG reaches a maximum voltage of 10.4 V at a flow rate of 1300 mL/min, representing a 1.9-fold increase compared to the pristine PVDF-based TENG. It also attained a maximum instantaneous power density of 50.6 μW/cm^2^. This energy output proved sufficient to illuminate 10 white LEDs. Furthermore, a correlation between the output electrical signals and water pulsating flow was established based on the amplitude and period of the output signals. Through regression analysis, a strong linear relationship was observed between the amplitude of the voltage and the flow rate, exhibiting high sensitivity ranging from 4.2 to 7.9 mV/mL.min. Simultaneously, an inversely proportional relationship was observed between the period of the pulsating voltage signal and the flow rate, with a constant proportionality of 74.284 s.mL/min. The high correlation coefficient R^2^, within the range [0.951, 0.998], underscores the accuracy of the proposed FL-TENG, showcasing its considerable potential as a self-powered pulsating flow sensor.

## 2. Experimental Section

### 2.1. Functionalized PVDF Membrane and FL-TENG Device Fabrication

**Figure 1:**
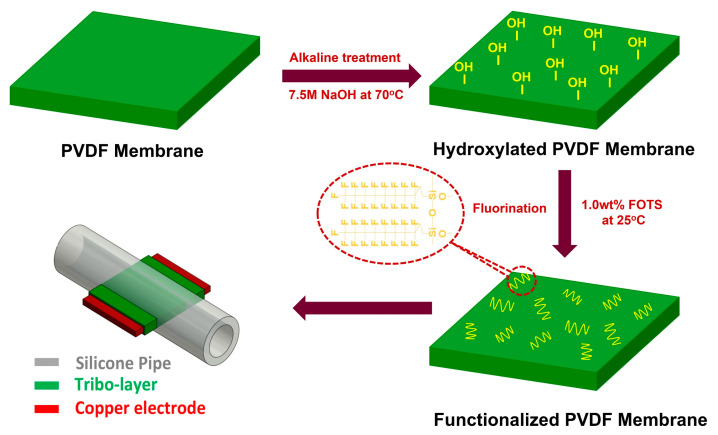
Schematic diagram of the procedure for functionalizing the PVDF membrane and diagram description of the FL-TENG device.

Figure 1 illustrates the procedural steps involved in transforming the functionalized PVDF membrane into the fabrication of the FL-TENG device. Initially, a PVDF membrane (50 µm, Sigma-Aldrich, St. Louis, MO, USA ) underwent treatment with an alkaline solution (7.5 M NaOH) for a duration of 3 hours at 70 °C to induce hydroxyl functionality. Subsequently, the hydroxylated PVDF membrane underwent fluorination by immersing it in a solution of 1H,1H,2H,2H-Perfluorooctyltriethoxysilane (FOTS, 98%, Sigma-Aldrich) with a concentration of 1.0 wt% for 24 h, resulting in the formation of the F-PVDF membrane. 

The sketch of a typical FL-TENG device, comprising a silicon pipe, a copper electrode, and a FL-PVDF membrane, is shown in Figure 1. The silicon pipes vary in inner diameters, specifically 3 mm, 5 mm, and 8 mm, denoted as 3 mm-pipe, 5 mm-pipe, and 8 mm-pipe, respectively. The width of the F-PVDF membrane corresponds to the inner diameter of the silicon pipe, determined by its size. It is important to note that the mentioned width pertains specifically to the contact area of the F-PVDF membrane inside the pipe. A copper electrode, with a thickness of 200 µm, is wrapped by the F-PVDF membrane and affixed at the center of the cross-sectional area of the pipe. For a visual representation, a real photograph of the FL-TENG device featuring an 8 mm-pipe is presented in Figure S1 (Supplementray Materials).

### 2.2. Characterization and Measurements

The surface structures of diverse membranes were examined using a JSM-7600 FE-SEM from JEOL Ltd., Tokyo, Japan. For the analysis of the chemical composition of these membranes, Fourier transform infrared (FTIR) analysis was conducted, using the Varian 640-IR FTIR Spectrometer, Varian Inc., Palo Alto, CA, USA. Additionally, an atomic force microscope (AFM) (MFP-3D Stand Alone AFM, Oxford Instruments, Abingdon, UK) was employed to analyze the surface roughness of the membranes. To assess hydrophobicity, the water contact angle of the membrane was measured using SmartDrop (FemtoFAB, Waltham, MA, USA ). The dielectric characteristics of the membranes were determined using an impedance analyzer, specifically the 3522-50 LCR Meter from Hioki Nagano, Japan, with a frequency range spanning from 1 to 10^7^ Hz.

**Figure 2:**
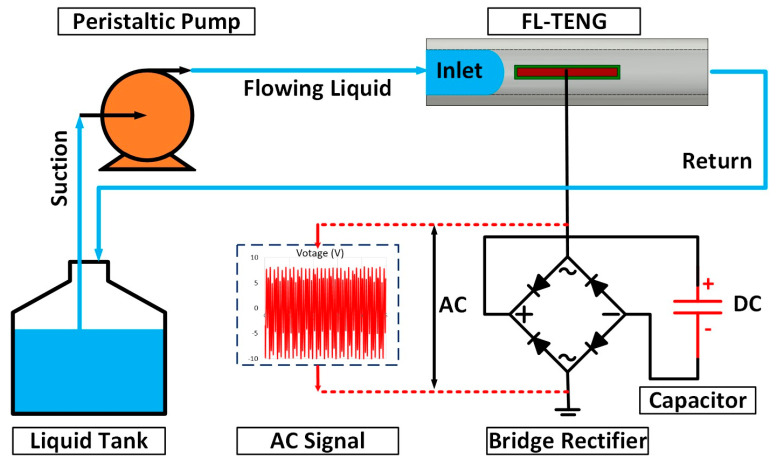
Schematic diagram of the experimental set up of the FL-TENG device.

The method of Structured Analysis and Design Technique was employed to delineate the function of the proposed FL-TENG system, as illustrated in Figure 2 and Video S1 (Supplementary Materials). For measuring the generated output, a digit graphical sampling multimeter was utilized (Keithley DMM7510, Keithley, OH, USA ). To facilitate this assessment, the AC-generated voltage underwent conversion to DC using a bridge rectifier. The output power was then stored in capacitors with varying capacitance values. The typical peristaltic pump utilized in this system featured a rotor equipped with multiple rollers (specifically, a three-roller pump) attached to an external flexible tube. During rotation of the rotor, the compression of a section of the tube resulted in closure, forcing the fluid to move through the tube, thus facilitating the pulsatile flow.

## 3. Results and Discussions

### 3.1. Working Mechanisms

**Figure 3:**
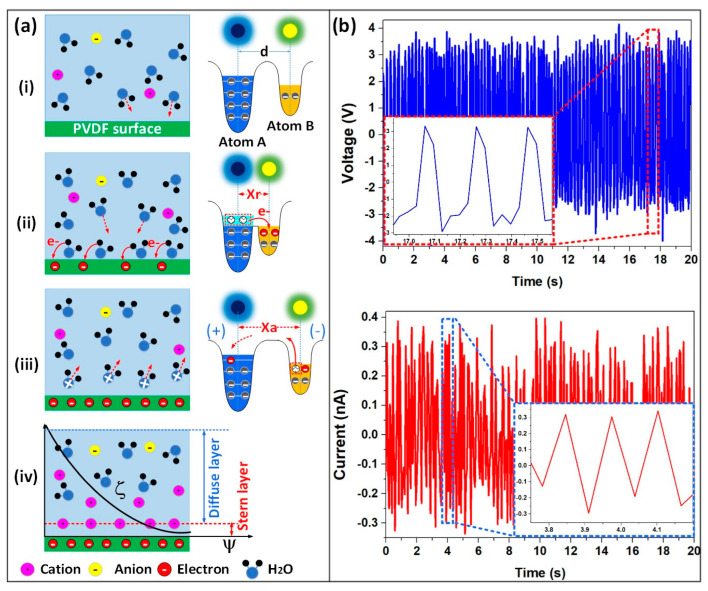
(**a**) Schematic diagram of contact electrification and the forming of EDL. (**b**) Output electrical signals of the SE-WTENG when water flows through the cell at a flow rate of 390 cc/min.

The working principle of the FL-TENG is based on the formation of an electric double layer (EDL) at the interface between water and the F-PVDF surface. Prior to contact, ions in water do not directly interact with those on the F-PVDF surface. However, upon contact, a bond is established with an overlap in electron clouds, creating an equilibrium distance between two atoms (Figure 3a(i)). The pressure from the water flow induces electron clouds to overlap when water ions impact the surface of the F-PVDF membrane, resulting in an interatomic distance shorter than the equilibrium distance (X_r_ < d). This facilitates electron transfer between water molecules and atoms on the F-PVDF surface (Figure 3a(ii)). Simultaneously, ionization reactions may occur on the solid surface, leading to both electron and ion transfer in the water–solid contact electrification (CE). The dominance of electron transfer in the CE is attributed to the hydrophobic of the triboelectric surface [47]. In the next stage, due to the pressure flow, water molecules adjacent to the F-PVDF surface are pushed away, increasing the interatomic distance (X_a_ > d). This diminishes the electron clouds’ overlap, breaking the formed bonds. The transferred electrons then remain on the F-PVDF surface as static charge, creating a negatively charged layer on the F-PVDF surface. The charged water molecules become freely migrating ions (Figure 3a(iii)). Then, as shown in Figure 3a(iv), the loosely distributed positive ions in water are attracted to adsorb onto the F-PVDF surface through electrostatic interactions, forming an EDL. As the flow carries away adsorbed ions on the F-PVDF surface, more charges are transferred across the interface to replenish the EDL. The formation of the EDL is likely a result of contact electrification stemming from electron transfer at the water–solid interface [14]. Due to electrostatic induction in the electrode, electrons alternatively flow between the electrode and the ground through the external circuit. Figure 3b illustrates the voltage and current of the FL-TENG, along with typical signals in inset images, measured at a water flow rate of 390 mL/min.

### 3.2. Electrical Output Characteristics

**Figure 4:**
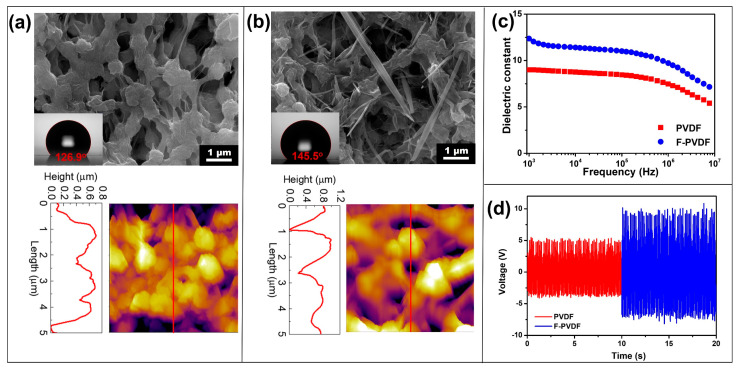
FE-SEM and AFM images of (**a**) PVDF membrane and (**b**) F-PVDF membrane; the inset image shows a contact angle; (**c**) frequency dependence of dielectric constant, and (**d**) output current of PVDF and F-PVDF-based TENG.

The analysis of the surface morphology of PVDF and F-PVDF membranes was conducted using FE-SEM and AFM, as illustrated in Figure 4a,b. The FE-SEM image reveals that the PVDF surface has a highly porous structure with evenly distributed pores ranging from 400nm to 600 nm and smooth pore walls. In contrast, the porosity of the F-PVDF membrane decreases due to the hydroxyl surface functionality process. The AFM images show a significant increase in the root mean square roughness (Rq) of the membrane, from 136 nm to 183 nm, after functionalization. This increase enhances the water contact angle of the membrane [48,49]. Moreover, the introduction of fluorine in FOTS, the most electronegative element, enhances polarizability and dipole moment, thereby manifesting superior hydrophobicity and dielectric constant properties [50,51]. The obvious increase in the water contact angle results is evident, surging from 126.3° to 145.5°. Exploring the dielectric constant of the PVDF and F-PVDF membrane, particularly in a frequency-dependent manner at room temperature, reveals a remarkable disparity (Figure 4c). Notably, at a frequency of 10^3^, the dielectric constant of the F-PVDF membrane is approximately 12.3, which is 35% higher than that of the PVDF membrane. To assess the impact of functionalization on FL-TENG output performance, the output voltage of PVDF and F-PVDF-based TENG were investigated. As anticipated, the F-PVDF-based TENG exhibits a notable voltage of 10.7 V, showcasing a remarkable 1.9-fold increase compared to the PVDF-based TENG. These results highlight that the F-PVDF membrane enhances the output performance of the FL-TENG, making it advantageous for use in self-powered pulsating flow sensors.

**Figure 5:**
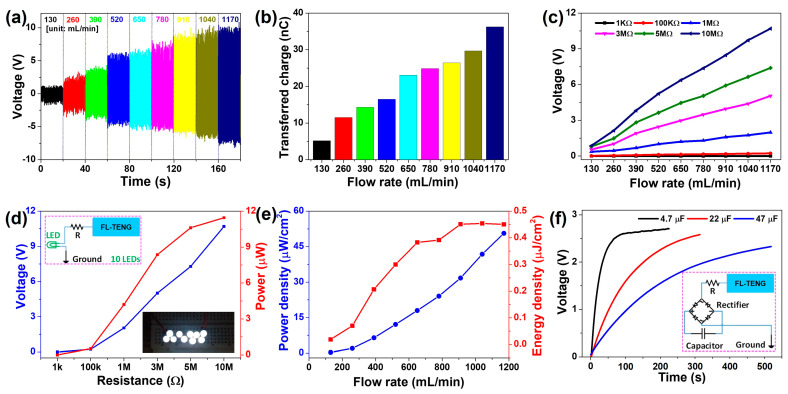
(**a**) Voltage and (**b**) transferred charge of the F-PVDF-based TENG depending on the flow rate (Different colors correspond to different flow rates shown as in the x-axis), (**c**) comparison of voltages measured at different resistances and flow rates, (**d**) voltage and power, and (**e**) power density and energy density of the F-PVDF-based TENG, measured at various resistances from 1 kW to 10 MW at a flow rate of 1170 mL/min, (**f**) charging of 4.7μF, 22μF, and 47μF capacitor by F-PVDF-based TENG.

To explore the potential application of the F-PVDF-based TENG, performance assessments were conducted using DI water, and an 8 mm-pipe was utilized to evaluate the performance of the FL-TENG. Real-time measurements, depicted in Figure 5a, were taken with different water flow rates. Evidently, as the flow rate increases, the shear force exerted by the flow on the F-PVDF surface rises, leading to the generation of more charge. The result demonstrates a consistent increase in voltage with the flow rate, reaching a maximum voltage of 10.2 V at a 1170 mL/min flow rate. Furthermore, electrostatic charge transfer, as illustrated in Figure 5b, can be determined using the following equation:

(1) $$Q_{c}=\frac{1}{R}\int_{t_{1}}^{t_{2}}Vdt$$

where Q_c_ is the electrostatic charge transfer, R is the electric resistance, V is the voltage, and t_1_ and t_2_ are the related times. According to this graph, the F-PVDF-based TENG can generate a charge transfer of 5.2 nC at a 130 mL/min flow rate, reaching a maximum value of approximately 36.3 nC at 1170 mL/min. Moreover, Figure 5c illustrates the relationship between flow rate and voltage, incorporating various resistors in the external circuit (1 kΩ to 10 MΩ). Following that, instantaneous power was calculated and reached 11.5 μW at a 1170 mL/min flow rate (Figure 5d). Remarkably, this power is sufficient to directly illuminate a series of 10 white LEDs, as shown in the inset figures and Video S2 (Supplementary Materials). Correspondingly, Figure 5e presents the calculations for the power density and energy density of the F-PVDF-based TENG. At a flow rate of 1170 mL/min, the FL-TENG demonstrates a power density of 50.6 µW/cm^2^ and an energy density of 0.45 µJ/cm^2^. Consequently, a bridge rectifier is introduced into the external circuit to convert the output electricity into direct current (DC) for charging the capacitor, as illustrated in the inset figure of Figure 5f. Various capacitors (4.7 µF, 22 µF, and 47 µF) are utilized to measure the charging capability of the FL-TENG under a flow rate of 1170 mL/min. A 4.7 µF capacitor charges to 2.6 V in about 90 s, whereas a 47 µF capacitor takes 500 s to charge to 2.3 V. These findings indicate the significant potential of the FL-TENG for application as a self-powered device.

### 3.3. Application in Self-Powered Flow Sensor

**Figure 6:**
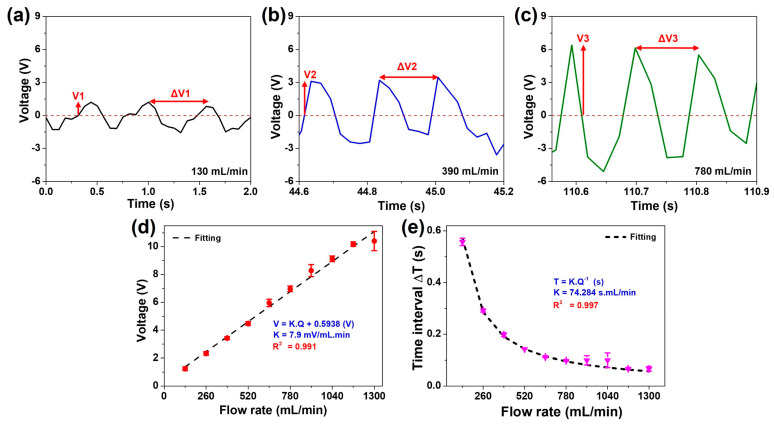
Electrical performance of F-PVDF-based TENG, depending on water flow conditions at a flow rate of (**a**) 130, (**b**) 390, and (**c**) 780 mL/min; regression analyses of the electrical response of FL-TENG based on (**d**) voltage and (**e**) time interval between two voltage peaks with different flow rates.

The objective of this study is to develop a self-powered sensor capable of detecting pulsating water flow within a millimeter-scale by monitoring the electrical response of the FL-TENG. Figure 6a–c shows typical output signals obtained at flow rates of 130, 390, and 780 mL/min from the experimental results of Figure 5a. In general, there are two signals that characterize the pulsating flow rate: amplitude, represented by the output voltage, and a period, represented by the time interval between two voltage peaks. As depicted in Figure 6a, at a flow rate of 130 mL/min, the F-PVDF-based TENG generates a voltage (V_1_) of 1.24 V, with a time interval between two peaks (ΔT_1_) measured as 0.501 s. Upon increasing the flow rate to 390 mL/min, the output voltage (V_2_) also rises, reaching values of 3.72 V. Subsequently, at a flow rate of 780 mL/min, V_3_ attains values of 6.21 V. In contrast, the time interval between two voltage peaks decreased with an increase in flow rate, yielding values ΔT_2_ of 0.173 s and ΔT_3_ of 0.105 s. It is evident that at lower flow rates, the TENG produces a relatively lower peak voltage with longer time intervals, whereas at higher flow rates, the peak voltage is higher, but the time interval is comparatively shorter.

The regression analysis is carried out on the electrical signal output concerning the flow rate to uncover the relationship between the pulsed electrical response and pulsating flow. Illustrated in Figure 6d,e, it is evident that the association between the voltage and the flow rate is a robust linear relationship, whereas the time interval between two voltage peaks demonstrates an inverse proportionality to the flow rate. The regression curves for these relationships are respectively delineated by:

(2) $$V=K_{v}\cdot Q+V_{0}$$

(3) $$T=\frac{K_{t}}{Q}$$

where V is the peak voltage (V), T is the time interval between two peaks of voltage (s), Q is the flow rate (mL/min), K_v_ and K_t_ are the constants of proportionality (or sensitivity), and V_0_ is the V-interpret. Consequently, an amplitude sensitivity (K_v_) of 7.9 mV/mL.min is attained for the voltage signal, accompanied by a high coefficient of determination R^2^ value of 0.991. Simultaneously, the proportionality constant (K_t_) for the period of time of the output signal is equal to 74.284 s.mL/min, corresponding to an R^2^ value of 0.997. These elevated sensitivities and high coefficients of determination affirm an exceptional accuracy of measurement, validating the estimation of flow rate through the monitoring of the output electrical signal.

Moreover, it is interesting to draw a comparison between the correlation of flow rate with the period of the output electrical signal and the relationship between flow rate and the period of pulsating flow produced by the pump. This comparative analysis serves as evidence for the potential application of the FL-TENG. Typically, the three-roller peristaltic pump generates the pulsating flow through the interaction among the rollers and the flexible tube. A water pillow is formed between two rollers, advancing along the tube in the direction of the revolving rotor, and is subsequently propelled into the discharge outlet (Video S3, Supplementary Materials). The operation involves alternating compression, squeezing, and release of the tube, enabling the generation of a pulsating flow of water during a single roller-step. Consequently, three pulses of flow are produced within one revolution of the rotor. Therefore, the period (T’ in seconds) of the pulsating flow, referred to as the roller-step period, can be calculated by:

(4) $$T^{\prime }=\frac{60}{N\cdot n}$$

where N is the pump speed (rpm), and n is the number of rollers (n = 3). Moreover, the flow rate Q’ in (L/min) delivered by a roller-step is given by:

(5) $$Q^{\prime }=\frac{1}{2}{\pi^{2}d}^{2}N\cdot r$$

where d is the inner diameter of the tube (mm), and r is the radius of the rotation(mm) that is measured from the rotor axis to the center of the rollers. The calculation of the pump is plotted in Figure S2 (Supplementary Materials). From the above equations, the relationship between the period of pulsating flow and flow rate can be determined by:

(6) $$T^{\prime }=\frac{10\cdot {\pi^{2}d}^{2}N\cdot r}{Q^{\prime }}$$

After applying the value of all the variables, this equation becomes:

(7) $$T^{\prime }=\frac{74.284}{Q}$$

Undoubtedly, there exists an inverse proportionality between the flow rate and the period of pulsating flow. Notably, this equation aligns with the regression equation established between the period of output electrical signal and the flow rate in the above discussion. By considering the analysis of both amplitude (voltage) and period (time interval) measurements of the output voltage, it is evident that the FL-TENG device has the capability to provide detailed information about the pulsating flow.

**Figure 7:**
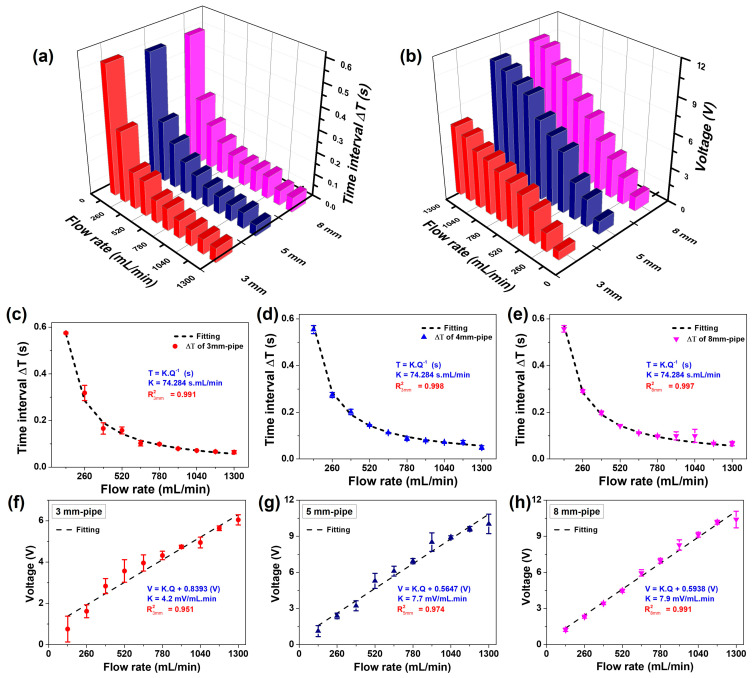
(**a**) Time interval between two voltage peaks and (**b**) output voltage, depending on the flow rate with different FL-TENG pipe sizes; regression analyses of the electrical response of (**c**) 3 mm-pipe, (**d**) 5 mm-pipe, (**e**) 8 mm-pipe-FL-TENG based on time interval between two voltage peaks and flow rates; regression analyses of the electrical response of (**f**) 3 mm-pipe, (**g**) 5 mm-pipe, and (**h**) 8 mm-pipe-FLTENG based on voltage and flow rate.

On the other hand, to demonstrate the precision of the FL-TENG in millimeter-scale measurements, various F-PVDF-based TENGs are fabricated with the 3 mm-pipe, 5 mm-pipe, and 8 mm-pipe (Figure 7a,b). The corresponding electrical responses, covering a range of flow rates from 130 to 1300 mL/min, are presented in Figure S3 (Supplementary Materials). Figure 7c–e presents the relationship between the measured time interval of two voltage peaks and the flow rate. This plot reveals that the associated time interval is prolonged at lower flow rates and shorter at higher flow rates. The regression analysis between the time interval and flow rate for all three FL-TENGs yields the same fitting curve. A constant proportionality of 74.284 s.mL/min is obtained, accompanied by a high correlation coefficient of R^2^ values of 0.991, 0.998, and 0.997, respectively, for the 3 mm-pipe, 5 mm-pipe, and 8 mm-pipe. It indicates a great proportional relationship between the period of the voltage signal and the flow rate. This highlights a robust proportional connection between the period of the voltage signal and the flow rate, irrespective of the diameter of the pipe. 

The regression analyses of the electrical performance of the FL-TENG based on voltage and flow rate are illustrated in Figure 7f–h. In the case of the 8 mm-pipe, a linear relationship is obtained with a sensitivity of 7.9 mV/mL.min and a correlation coefficient R^2^ of 0.991, as discussed previously. Similarly, sensitivities of 7.7 and 4.2 mV/mL.min are identified for the 5 mm-pipe and 3 mm-pipe, corresponding to R^2^ values of 0.974 and 0.951. Although the sensitivity of the 3 mm-pipe is significantly smaller than these two FL-TENGs, it still exhibits a high linear relationship, confirming the suitability of the FL-TENG for accurate millimeter-scale flow rate measurement. These discussions demonstrate that monitoring the voltage signals of the F-PVDF-based TENG allows for the determination of the water pulsating flow rate.

In practical applications, the stability and durability of a sensing device are crucial for obtaining correct and accurate measurements. To assess the stability of the F-PVDF-based TENG, experiments are conducted one month apart to validate the stability of the output signal. As illustrated in Figure S4 (Supplementary Materials), the FL-TENG exhibits a minimal change in electrical performance, indicating good working durability and stability. 

## 4. Conclusions

In summary, a novel self-powered pulsating flow sensor has been developed using an FL-TENG, where the determination of pulsating flow involves monitoring the output electrical characteristics, specifically the amplitude and period of the pulsating voltage signal. The performance of the FL-TENG is significantly enhanced by increasing the transferred charge density of the triboelectric layer. The F-PVDF-based TENG achieves a maximum voltage of 10.4 V at a 1300 mL/min flow rate, representing a 1.9-fold increase compared to the pristine PVDF-based TENG. Moreover, the results from regression analysis indicate a linear relationship between the output voltage and the flow rate, while the time interval between two voltage peaks is inversely proportional to the flow rate. With high sensitivity and a coefficient of determination ranging from 0.951 to 0.998, the electrical performance underscores the suitability of the FL-TENG for accurate pulsating flow measurement on a millimeter-scale. Furthermore, the device can function as a power source, contributing to the advancement of a self-powered pulsating flow sensor. The F-PVDF-based TENG exhibits notable advantages, including low cost, a straightforward structure, easy installation, reliability, and effectiveness in the millimeter-scale pipelines. 

## Data Availability

The authors confirm that the data supporting the findings of this study are available within the article.

## Supplementary Materials

The following supporting information can be downloaded at: https://www.mdpi.com/article/10.3390/polym16040536/s1, Figure S1: Real photograph of FL-TENG testbench; Figure S2: Characteristics of the water pump; Figure S3: Output voltage and current of the FL-TENG at a variable flow rate by different sizes of silicone pipes (a,b) 3 mm-pipe, (c,d) 5 mm-pipe, and (e,f) 8 mm-pipe; Figure S4: Stability and durability of the FL-TENG. (a,c) Output voltage measured at 390 and 650 mL/min of the FL-TENG at two moments with one month apart. (b,d) Stability test of the FL-TENG for 300 s at a flow rate of 390 and 650 mL/min; Figure S5: FTIR spectra of PVDF and F-PVDF membrane. Video S1: Experimental testbench; Video S2: Demonstration of FL-TENG performance: 10 white LEDs are directly lighted up; Video S3: Demonstration of the three-roller peristaltic pump.
