# Supplementary material for: Flowing Liquid-Based Triboelectric Nanogenerator Performance Enhancement with Functionalized Polyvinylidene Fluoride Membrane for Self-Powered Pulsating Flow Sensing Application
Source: Polymers (Basel). 2024 Feb 16;16(4):536. doi: 10.3390/polym16040536 (PMC10891804; doi:10.3390/polym16040536)
Supplement: Supplementary file 1 [file polymers-16-00536-s001.zip › polymers-2847047-supplementary.docx]

Supplementary Material

Flowing liquid-based triboelectric nanogenerator performance enhancement by functionalized polyvinylidene fluoride membrane for self-powered pulsating flow sensing application

Duy Linh Vu^1^, Quang Tan Nguyen^2^, Pil Seung Chung^1,3,*^ and Kyoung Kwan Ahn^2,^*

|  |
| --- |

^1^ Department of Nanoscience and Engineering, Inje University, 197 Inje-ro, Gimhae-si, Gyeongsangnamdo 50834, Republic of Korea

^2^ School of Mechanical Engineering, University of Ulsan, 93 Daehak-ro, Nam-gu, Ulsan, 44610, South Korea

^3^ Department of Energy Engineering, Inje University, 197 Inje-ro, Gimhae-si, Gyeongsangnamdo 50834, Republic of Korea

***** Correspondence: pschung01@inje.ac.kr (Pil Seung Chung) and kkahn@ulsan.ac.kr (Kyoung Kwan Ahn).

**
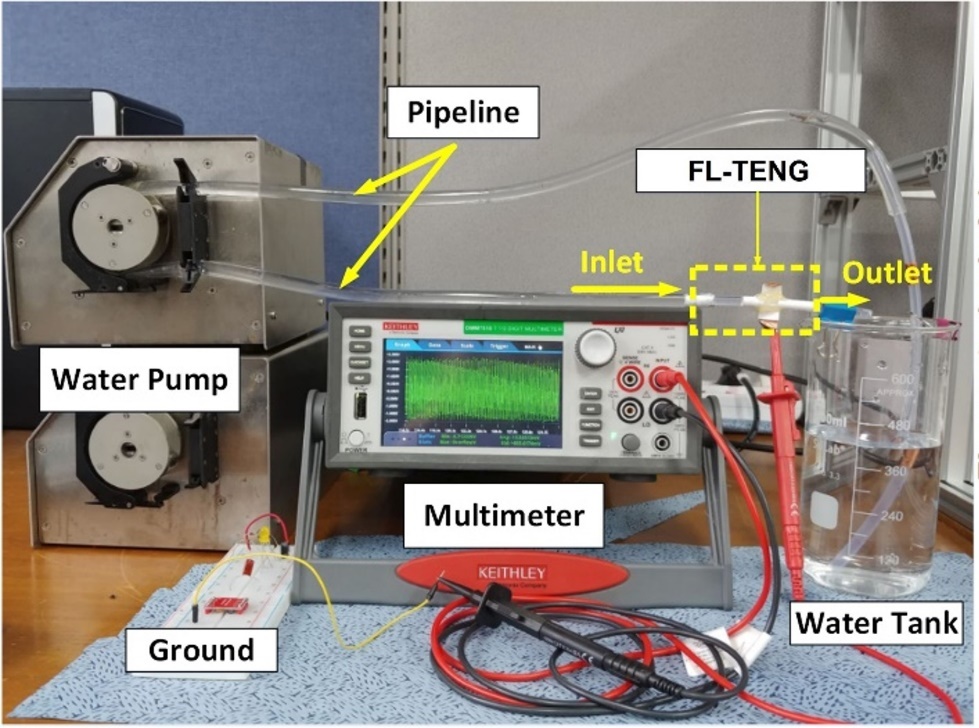
**

**Figure S1.** Real photograph of FL-TENG testbench.


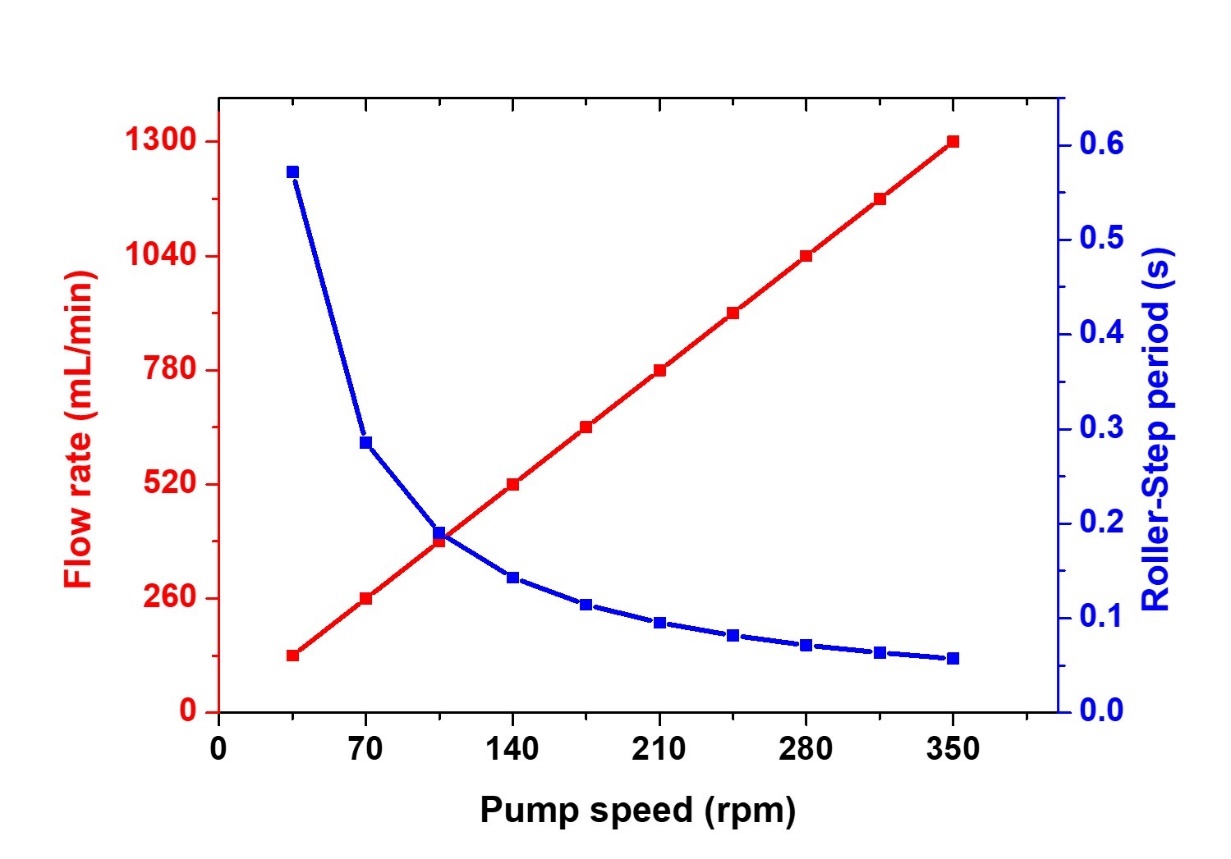


**Figure S2.** Characteristics of the water pump


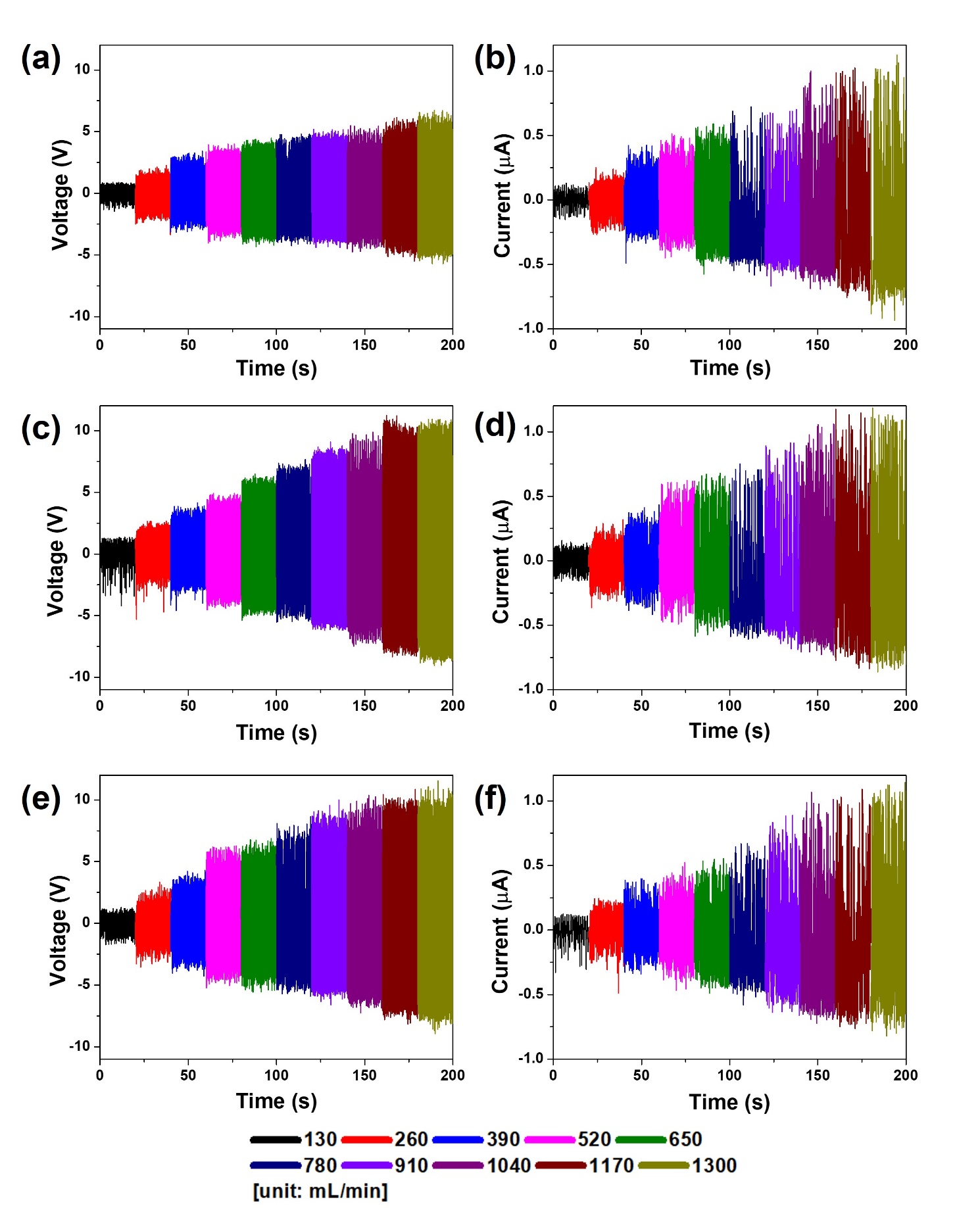


**Figure S3:** Output voltage and current of the FL-TENG at a variable flow rate by different sizes of silicone pipe (a, b) 3 mm-pipe, (c, d) 5 mm-pipe, and (e, f) 8 mm-pipe.


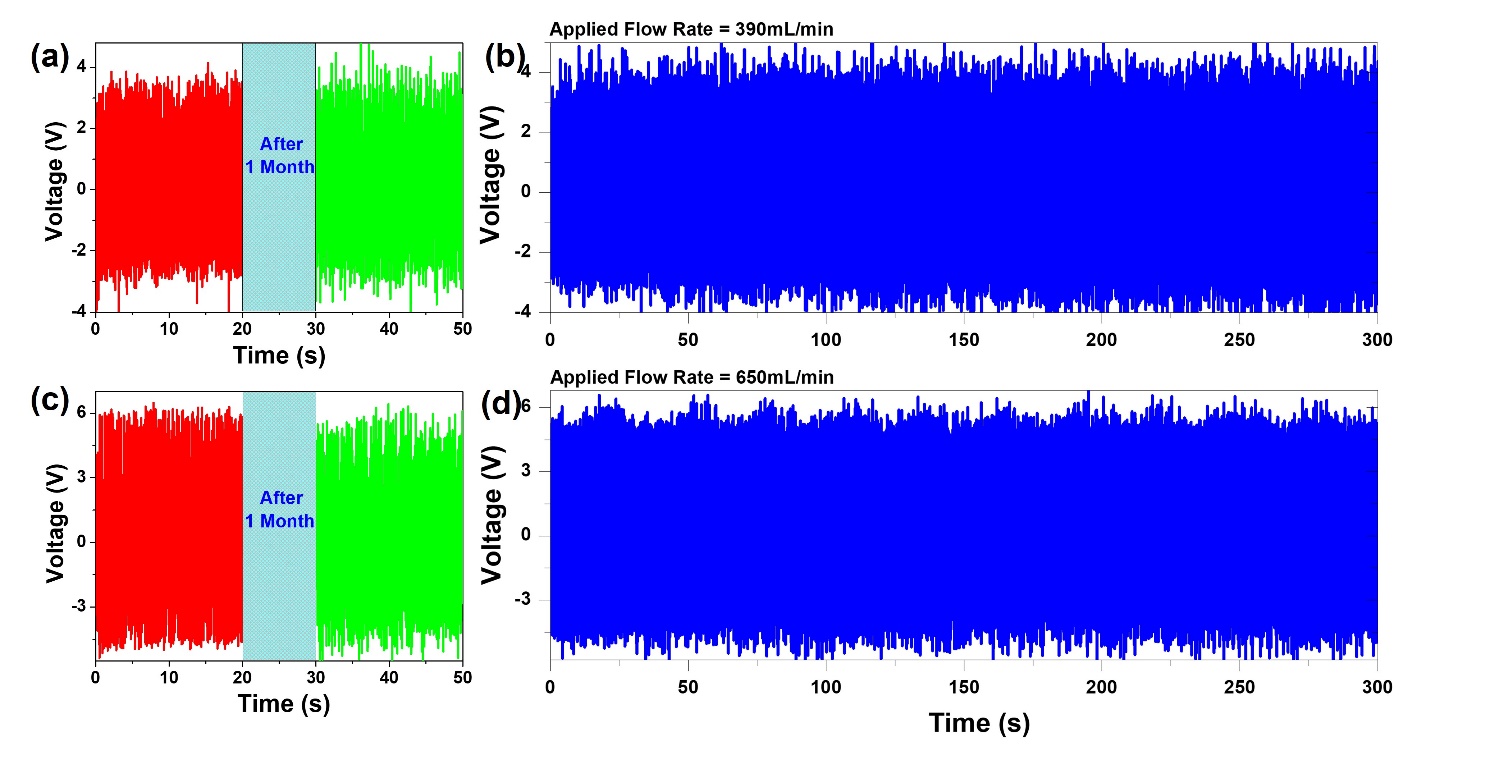


**Figure S4:** Stability and durability of the FL-TENG. (a, c) Output voltage measured at 390 and 650 mL/min of the FL-TENG at two moments with one month apart. (b, d) stability test of the FL-TENG for 300 seconds at a flow rate of 390 and 650 mL/min.

**Video S1**: Experimental testbench

**Video S2**: Demonstration of FL-TENG performance: 10 white LEDs are directly lighted up.

**Video S3:** Demonstration working of the three-roller peristaltic pump.
